# Editorial: Ion channels and transporters as drug targets in immune disorders

**DOI:** 10.3389/fphar.2024.1439156

**Published:** 2024-06-18

**Authors:** Vaibhavkumar S. Gawali, Yogesh B. Narkhede, Sachin Kumar

**Affiliations:** ^1^ Frontage Laboratories, Concord, OH, United States; ^2^ University of Notre Dame, Notre Dame, IN, United States; ^3^ Central Drug Research Institute, Lucknow, India

**Keywords:** ion channels, immune cells, inflammation, channelopathies, pharmacology, immune disorders, molecular drug targets

Ion channels and transporters regulate critical functions in immune cells such as controlling effector functions, intracellular signaling and cell physiology. Defective functions of ion channels in immune cells often lead to loss of cellular functions and results in immune disorders. Immunology research has gained momentum since the emergence of COVID-19 pandemic and novel drug targets are being explored to develop therapies targeting various immune disorders including COVID-19.

Recent exploration of crystal structures of various ion channels offered increased understanding about structure-function relationships of therapeutically important ion channels. Technological advances in patch-clamp electrophysiology have enabled researchers to characterize multiple ion channels and screen novel drugs at much faster rate. The changing landscape of academic research settings from basic science to translational studies has given researchers an improved access to patient blood samples and opportunities to study ion channels and transporters in immune cells and related disorders.

The present Research Topic, which is jointly issued in Frontiers in Immunology and Frontiers in Pharmacology, contains a Research Topic of seven articles focusing on the promising role of ion channels and transporters as therapeutic drug targets in multiple immune disorders such as COVID-19, rheumatoid arthritis. The Research Topic offers three original research articles and four reviews highlighting the key ion channels as druggable targets in acute and chronic immune disorders that currently lack effective therapies.

Three articles in our Research Topic focus on K^+^ channels in T cells involved in inflammatory immune disorders and novel therapies for treatment of these disorders. Chimote et al. studied the cytotoxic T lymphocytes of severely ill COVID-19 patients treated with dexamethasone and identified the critical role of voltage-gated potassium channel (Kv1.3) as potential drug target in COVID-19 treatment. This translational study revealed that dexamethasone inhibited Kv1.3 channel function in T cells and reduced the cytokine storm in severe COVID-19 patients. This study aligns with previous findings confirming that Kv1.3 has emerged as an attractive drug target in controlling inflammation and related disorders. A detailed review from More et al., on the role of Kv1.3 channels along with Ca^2+^ activated potassium channels KCa3.1 in inflammatory disorders such as rheumatoid arthritis highlights the therapeutic importance of Kv1.3 and KCa3.1 channels in autoimmune disorders. This review also summarizes the progress made towards developing novel therapies targeting Kv1.3 and KCa3.1 channels.

Set of review articles focusing on TRPV1 and NALCN ion channels in immune cells and highlight their therapeutic importance in immune disorders. An intriguing review from Qu et al., describes the pivotal role of TRPV1 channels in the pathology of rheumatoid arthritis. This review provides a comprehensive summary of involvement of TRPV1 channels expressed in variety of immune cells such as T cell, macrophages, dendritic cells, microglia impacting the signaling pathways in disease biology of rheumatoid arthritis.

In their mini-review, Zhang and Wei, summarize recent updates in the literature on the emerging role of sodium leak channels (NALCN) in neuropathic and inflammatory pain sensation. NALCN belongs to the family of sodium channels and is overexpressed in neuro-immune cells such as astrocytes and oligodendrocytes and peripheral nervous systems such as DRGs. The article highlights the need for NALCN specific blockers to develop novel analgesics. Furthermore, a short review from Wu et al. provides an update on the role of various ion channels in macrophages involved in inflammatory disease such as atherosclerosis. The review highlights key cation and anion ion channels expressed in macrophages and are involved in the pathogenesis of atherosclerosis.

Another two research articles report novel therapeutic developments targeting TRPM4 and Piezo1 channels. Arullampalam et al. identified the species-specific effects of cation channel TRPM4 small molecule inhibitors. Key findings in this research article provide researchers 2 new investigational tools and potential alternative to traditionally used small molecule inhibitor 9-phenathrol to study TRPM4 channels, therapeutically important target in cardiac and immune disorders.

Last but not least, a study on mechanically activated Piezo1 channels from Zhou et al. reveals insightful mechanisms by which mutations influence Piezo1 channel function responsible for causing immune disorders such as generalize lymphatic dysplasia. This study provides experimental evidence of rescuing mutation induced defects in protein trafficking by using clinically used protease inhibitor Bortezomib. Their findings suggest a futuristic approach for developing new therapies in combination with a precision medicine based approach to treat Piezo1 channelopathies.

In summary, this article Research Topic highlights the importance of ion channels as drug targets in immune disorders.

